# Side Population Cells as Prototype of Chemoresistant, Tumor-Initiating Cells

**DOI:** 10.1155/2013/517237

**Published:** 2013-11-04

**Authors:** Vinitha Richard, Madhumathy G. Nair, T. R. Santhosh Kumar, M. Radhakrishna Pillai

**Affiliations:** Cancer Research Program, Rajiv Gandhi Centre for Biotechnology, Thiruvananthapuram, Kerala 695014, India

## Abstract

Classically, isolation of CSCs from tumors exploits the detection of cell surface markers associated with normal stem cells. Invariable expression of these cell surface markers in almost all proliferating tumor cells that albeit impart specific functionality, the universality, and clinical credibility of CSC phenotype based on markers is still dubious. Side Population (SP) cells, as defined by Hoechst dye exclusion in flow cytometry, have been identified in many solid tumors and cell lines and the SP phenotype can be considered as an enriched source of stem cells as well as an alternative source for the isolation of cancer stem cells especially when molecular markers for stem cells are unknown. SP cells may be responsible for the maintenance and propagation of tumors and the proportion of SP cells may be a predictor of patient outcome. Several of these markers used in cell sorting have emerged as prognostic markers of disease progression though it is seen that the development of new CSC-targeted strategies is often hindered by poor understanding of their regulatory networks and functions. This review intends to appraise the experimental progress towards enhanced isolation and drug screening based on property of acquired chemoresistance of cancer stem cells.

## 1. Introduction

The fundamental problem of tumor recurrence and failing conventional therapies is largely due to the continuing presumption that human cancer cell populations are homogeneous and every cell in a tumor has indistinguishable tumorigenic potential. Recent experiments, however, insinuate that human tumors may not in fact be functionally homogeneous but comprise of a very small fraction of cells that possess actual tumorigenic potential [1, 2]. This scenario subsequently led to the postulation of the cancer stem cell hypothesis which puts forth that cancer cells have an hierarchical developmental structure in which only a fraction of cells termed cancer stem cells (CSCs) can proliferate indefinitely and form tumors [3]. One of the great advantages of the cancer stem cell hypothesis is that it also helps in understanding other cancer concepts such as minimal residual disease [4]. Cancers that follow the CSC model may as well undergo clonal evolution if more than one type of CSCs coexist or CSCs are under environmental selection [5]. Furthermore, series of genetic mutations impart one or another type of growth advantage instigating Darwinian evolution and survival of a group of stronger stem-like cancer cells overruling translation to malignancy [6]. Evidence that either stem or progenitor cells can act as targets for tumor initiation in a range of solid tumors have been exclusively reviewed by Visvader [7]. Substantiation of this hypothesis has gradually gathered pace over the past few years opening up the reality that design of current treatment strategies may have overlooked these pivotal cells and their molecular networks that hold the key to tumor recurrence and relapse. 

Understanding the molecular and cellular basis of tumor heterogeneity both in hematological and solid malignancies and related treatment resilience requires accurate discrimination of tumor propagating stem-like cells from the nonmalignant cells. This review focuses on the experimental advances made in the direction of uncovering CSCs in multiple tumor types and elucidates their role in enhanced chemo-resistance and metastatic potentials. We also discuss herein the major regulatory networks governing CSC-mediated chemoresistance and CSC-based drug screening assays leading to effective futuristic modes of therapeutic interventions. 

## 2. Proof of CSC Concept—The Assays

Self-renewal and lineage capacity are the hallmarks of any stem cell. Therefore, as with normal stem cells, assays for cancer stem cell activity need to be evaluated for their potential to show both self-renewal and tumor propagation. Prospective isolations of CSC allow their direct comparison to normal stem/progenitors, revealing important information about CSC regulation, CSC origins, and disease pathogenesis. Purification of solid tumor-initiating cells (T-IC) has been difficult because of the universal expression pattern of most cell surface markers that are currently selected for cell sorting [8]. T-IC xenograft assays for primary human solid tumor tissue in nude mice pose the challenge of residual immune function, triggering host resistance mechanisms that will not permit single T-IC to be detected [9]. Xenotransplantation systems only measure the ability of a human tumor cell to grow in a permissive mouse niche and do not reflect the actual intrinsic behaviour *in vivo*. At present, there is no superior assay system to measure the tumorigenic potential of primary human tumor isolates [3, 10].

An ideal *in vitro* assay would be (a) quantitative, (b) highly specific and sensitive to detect CSCs even at low frequency, and (c) rapid. Several *in vitro* assays have been used to identify tumor stem cells, including sphere assays, serial colony-forming unit (CFU) assays (replating assays), and label-retention assays [3]. Expression of surface markers by CSCs may vary over time and/or by location [11]. Isolation of CSCs was based on functional activity when CSC cell surface phenotypes could not be applied to all cancers arising from the same tissue type. Investigators have FACS-purified and functionally characterized human cancers based on aldehyde dehydrogenase (ALDH) expression, demonstrating that ALDH+ cells are enriched for CSCs in primary breast [12] and colon cancer [13]. In some cases, markers not previously identified on normal stem/progenitors have been used to isolate tumorigenic populations. ABCB5, a known chemoresistance mediator in melanoma, was expressed in only a minority of tumor cells (1.6–20.4%) and only ABCB5+ cells were capable of establishing primary grafts and serially transplanting disease in NOD/SCID mice [14].

**Figure 1:**
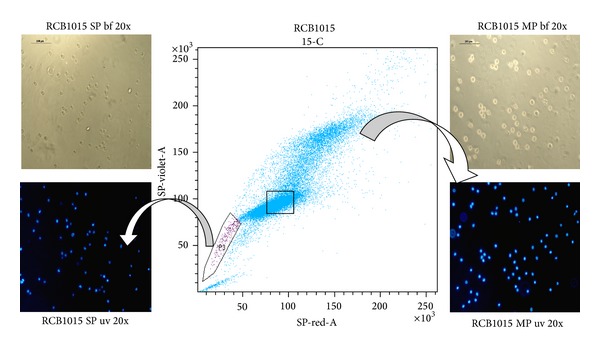
“Side population” phenotype in an oral squamous cell carcinoma cell line, RCB1015. The cell line was stained with Hoechst33342 and analyzed by flow cytometry. (SP-side population; MP-main population; bf-bright field image; uv-ultraviolet fluorescence; blue color- nuclear staining with Hoechst33342 dye).

An early method for stem cell isolation used the drug efflux property of stem cells. Most cells accumulate fluorescent dyes such as Hoechst 33342 and Rhodamine, but a subset of “dull cells” is often found and termed the “side population” (SP phenotype) (Figure 1). A large fraction of hematopoietic stem and tissue stem cells is in the SP fraction, and most of the cells in the SP fraction are stem cells (extensively reviewed in [15]). These cells maintain a high efflux capability for antimitotic drugs as well. The method was first described in murine bone marrow cells while displaying Hoechst fluorescence simultaneously at two emission wavelengths, wherein SP cells represented a small subset of cells that were enriched at least 1000-fold in HSC activity [16]. SP cells isolated from malignant gastric [17] and gallbladder carcinoma [18] cells have been shown to exhibit stem cell characteristics such as unlimited self-renewal, multipotent potential, and drug resistance [3].

 The SP cells are identified according to their ability to efflux the Hoechst dye at a higher pace than the remaining tumor cells termed the main population (non-SP). Moreover, the degree of efflux activity seems to correlate with the maturation state, such that cells displaying highest efflux activity are the most primitive in terms of differentiation potential. There is also a direct link between dye efflux and stem cell capacity, such that the cells at the lowest tip of SP phenotype (with the highest dye efflux and least amount of dye) exhibited the highest stem cell activity over the longest period of time [16]. T-ICs sorted in this method displayed self renewal capabilities *in vivo*; that is, the sorted cells generated a tumor mass in mice every time they were transplanted into a new mouse [19]. However, as with cell surface markers, possession of an SP phenotype is not a universal property of stem cells, and in some tissues, the SP fraction may not contain the stem cells. Indeed, combining SP determination with cell-surface marker phenotyping has lead to efficient and reliable characterization of one of the most pure and potent adult stem cell populations, the HSC subset. The SP assay has finally emerged as a promising method for identifying stem cell and progenitor populations in different tissues (Table 1), particularly in the absence of specific cell-surface markers [20].

## 3. Regulators of Multidrug Resistance in CSCs

**Figure 2:**
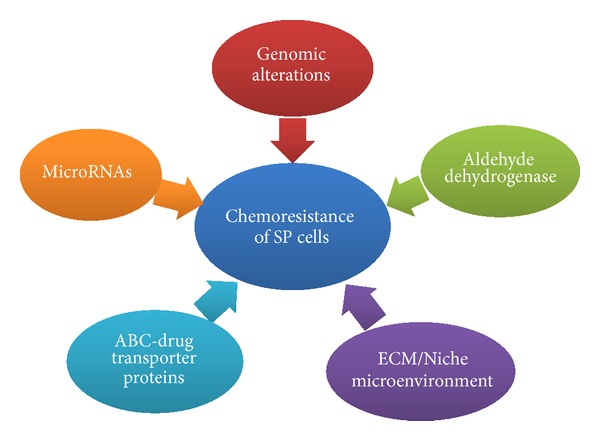
Prime instigators for chemoresistance in tumor stem cells. Factors contributing to acquired chemoresistance in cancer stem cells epitomized by side population phenotype across multiple tumor types.

In clinical scenario, many patients with solid tumors respond poorly to existing treatment regimens (including chemotherapy, radiation, and tumor-targeted agents) or relapse quickly after an initial remission. Several characteristics that make CSCs resistant to conventional chemo- and radiotherapy include high expression of drug transporters, relative cell cycle quiescence, high levels of DNA repair machinery, and resistance to apoptosis [15]. The potential for quiescence of cancer stem cells is also a potential concern, and these cells may be resistant to drugs even in the absence of transporter expression or activity. The nature of clinical drug resistance is multifactorial, involving alteration in drug targets, inactivation/detoxification of the drug, decreased drug uptake, increased drug efflux, and the dysregulation of apoptotic pathways [21]. ABC transporters are not the sole cause of drug resistance in CSCs; several other factors (Figure 2), such as the capacity of a stem cell for DNA repair and its quiescent state, may also have an impact on drug resistance in a tumor [22].

### 3.1. Drug Transporters

Amongst the three “ATP-binding cassette (ABC)” transporters ABCB1 (MDR1/P-glycoprotein), ABCC1, and ABCG2, (BCRP) had the highest expression in side population (SP) cells [23]. ABCG2 emerged as an important multidrug resistance protein because it confers cross-resistance to several structurally unrelated classes of cancer chemotherapeutic agents [24]. A recent discovery showed that Nrf2, an oxidative stress sensor, maintains the SP cell phenotype by upregulating ABCG2 expression through its direct interaction with an antioxidant response element [ARE] on the ABCG2 promoter [25]. The functional MDR1 conferred resistance to apoptosis induced by chemotherapeutic drugs in addition to a fundamental role in regulating cell death mediated by caspases. MDR1 has also been shown to confer resistance to cell lysis induced by activated complements [26]. This growing body of evidence implicates the importance of transporter proteins in the protection of the SP cells against a diverse range of cell death stimuli by functioning as an energy-dependent pump, which exports drugs out of drug-resistant mammalian cells, lowering the intracellular drug concentration to sublethal levels, thus acting as determinants of the chemoresistant phenotype of stem-like SP cells in several human malignancies. They also impart resistance to multiple chemotherapeutic drugs currently used in therapy (Table 2).

### 3.2. Genomic Instability

 Cancers in general exhibit extensive modifications in genome composition, ranging from subtle point mutations to dramatic gains and loss of genetic material (aneuploidy) [27]. The vast majority are likely to be passenger mutations [somatic mutations without functional consequences] that do not contribute to the development of cancer or confer any clonal growth advantage that often occur during cell division [28]. However, a small minority are critical drivers of tumorigenesis that would have occurred during growth of the cancer, conferring a growth advantage by positively selecting cancer cells in the microenvironment of tissue origin [28]. These alterations also render tumor cells the ability to evade growth-inhibitory signals, resulting in uncontrolled tumor growth. To affirm that genomic instability is involved in the induction of CSCs, studies have been conducted using side population (SP) cells in human nasopharyngeal carcinoma [NPC CNE-2] and CD133+ human neuroblastoma cells [29]. These cells were subjected to DNA damage by ultraviolet light and mitomycin C treatment resulting in an increase in SP fraction with overexpression of cell cycle regulators in NPC and neuroblastoma SKN-SH cells. An increase in the number of SP cells was also observed in recurrent tumor tissue as compared with the primary tumor in the same NPC patient [29]. Studies mention that DNA methylation could also be one of the reasons for acquired drug resistance. A total of 13/41 genes were consistently hypermethylated in cisplatin-resistant ovarian cancer cell line A2780 cell derivatives. Furthermore, 5/13 genes [ARMCX2, COL1A1, MDK, MEST, and MLH1] acquired methylation in SP cells isolated from drug-resistant ovarian cancer [30]. 

### 3.3. Signalling Pathways

Expression of EGFR and other erbB receptors are deregulated in many cancers [31] and a study on head and neck squamous cell carcinoma cell lines showed that activation of EGFR leads to increase in chemoresistant fraction of SP cells thereby playing a role in regulating cancer stem cells and tumorigenesis in such tumors [32]. IRESSA [Gefitinib-EGFR inhibitor] plus Vincristine treatment led to impairment of SP phenotype [32]. PI3K pathway is also said to be involved in regulating the SP phenotype. Recently, it was shown that a PI3K inhibitor LY294002 could inhibit all ABCG2, MDR1, and MRP1, the three classes of ABC transporters that were overexpressed in SP cells and multidrug resistance [33]. Treatment with Hedgehog [Hh] pathway inhibitor Cyclopamine or Cyclopamine and Paclitaxel decreased the SP population and expression levels of MDR1 efflux protein in prostate cancer cell lines [34]. Gene profiling studies and protein profiling have demonstrated an elevated expression of chemoresistance associated proteins in various types of cancers. A recent study on human stage II breast cancer specimens showed an increased expression of Fra-1 a member of the Fos transcription factor family, correlated with a reduced side population fraction [35]. The reverse was observed between reduced expression of Fra-1 and chemoresistance [35] which implies the existence of various regulatory proteins that could be tuning the chemoresistant side population cells.

### 3.4. MicroRNA

 Recent researches have shed light on the biological importance of miRNAs in the maintenance of side population phenotype. The mammalian genome encodes hundreds of MicroRNAs [miRNAs] that collectively affect the expression of about one-third of all genes. They are an abundant class of small nonprotein-coding RNAs that function as negative gene regulators. Recent evidence has shown that dysregulation of miRNA activity is associated with disease. miRNAs have been shown to repress the expression of important cancer-related genes and might prove useful in the diagnosis and treatment of cancer. Certain miRNAs have been shown to promote oncogenesis “oncomirs” or to repress it [36]. Recent work by Misawa et al. [37] has highlighted the role of microRNA-21 (miR-21) and its upstream regulator activator protein-I (AP-1) in sustenance of the chemoresistant SP cells. Treatment of the cells with the AP-1 inhibitor SP600125 attenuated miR-21 levels and increased topotecan sensitivity [37]. miR-Let-7 is downregulated in SP cells and the downregulation in turn makes let-7 lose the prospects to restrain Ras mRNA, leading to activation of p-Ras and p-ERK [38]. Upregulation of let-7 also leads to suppression of ER*α* and may be a promising strategy for the inhibition of the ER signaling pathway and for the elimination of cancer stem cells, thus aiding in the treatment of breast cancer [39]. Recently, miR200c and 34a have also been shown to modulate chemoresistance against Cyclopamine and paclitaxel in prostate cancer cell lines [34]. miR-328 is also important for the maintenance of SP phenotype. miR-328 overexpression reversed drug resistance and inhibited cell invasion of SP cells of colorectal cancer [40].

### 3.5. Aldehyde Dehydrogenase (ALDH)

Similar to Hoechst dye extruding SP cells, high expression of ALDH appears to be a marker for stem cells from many tissues [41] and methodology to isolate viable cells by ALDH activity using a fluorescent labeled aldehyde substrate [Aldefluor] is now available. Data from several groups suggest that Hoechst and ALDH may identify SP cells from a variety of malignancies, and combined use of ALDH and Hoechst efflux activity to isolate the quiescent stem cell fractions and may be particularly useful in those malignancies where little is known about the phenotypes associated with the differentiation program of the tissues of origin [42]. The elevated expression of ALDH in side population cells might be playing a role in rendering resistance to chemotherapeutic agents. Aldehyde dehydrogenase is a polymorphic enzyme responsible for the oxidation of aldehydes to carboxylic acids. These genes participate in a wide variety of biological processes including the detoxification of exogenously and endogenously generated aldehydes [43]. In normal stem cells, specifically the ALDH1 family mediates the synthesis of intracellular all-trans-retinoic acid that is required for the growth of the hematopoietic system and other tissues [44]. The role of ALDH is not limited to retinoic acid metabolism, as it is also involved in the detoxification. ALDH1 is cytosolic, ubiquitously distributed, and in particular confers resistance to anticancer drugs of the cyclophosphamide family by their detoxification [44]. ALDH family of enzymes are known markers of chemoresistance as evident from studies done on various types of cancers and tumour initiating cells [45, 46]. Hence, targeting SP cells should be considered as alternative models for high throughput drug screening that might aid in significantly improving existing therapeutic inconsistencies associated with chemoresistant tumor-initiating cells.

## 4. Renewal of Therapeutic Realization

Identification of cancer stem cells to date is based on tumorigenic potential in permissive conditions and not dependent on the actual fate of cells within patients under specific conditions [47]. Cancer cells with tumorigenic potential could be held transiently or permanently, by environmental, epigenetic, or immunological mechanisms from actually contributing to disease as elucidated in CSC shift hypothesis [8, 47]. Agents or compounds that show specific activity against SP cells will have a definite role after primary debulking therapy. Drugs have to be developed to target specifically these SP cells that have higher malignant potential with numerous getaway strategies from immune surveillance and attack ensuing minimal residual disease [MRD], metastasis, and recurrence. Various attempts have been developed in formulating such drugs that would transpire as a promising cure for cancer, particularly in combination with chemotherapy. Salinomycin was found to act as a potent inhibitor of multidrug resistance gp170, as evidenced through drug efflux assays in cancer cell lines overexpressing P-gp/MDR1 [48]. Since SP cells overexpress MDR1 it might serve as a potent inhibitor of SP cells as well. Curcumin has shown to inhibit the side population [SP] cells of rat C6 glioma cell line and is a dietary phytochemical with potential to target stem cell mediated tumor initiation and development [49]. A reactive oxygen species [ROS] generator Emodin (1,3,8-trihydroxy-6-methylanthraquinone), suppressed the function of ABCG2 via ROS-related mechanism. Emodin was also able to sensitize SP cells to cisplatin by the inhibition of expression of abcg2 [18]. The PI3K/mTOR inhibitor NVP-BEZ235 and PI3K inhibitor Wortmannin were capable of decreasing the SP fraction and the drug efflux along with a significant reduction in the levels of ABCG2 [50]. Low-molecular-weight heparin [LMWH] combined with cisplatin could overcome cisplatin-resistance and induced apoptosis in lung SP cells both *in vitro* and *in vivo* [51]. Tetrahydrocurcumin (THC), the ultimate metabolite of the curcumins *in vivo*, was found to be able to inhibit the function of P-gp, MRP1, and MXR and additional evidences also directed towards the development of curcumin I and THC as MDR modulator to use in combination with conventional chemotherapy [52]. Even though several strategies for targeting cancer stem cells have been proposed, a number of concerns related to efficacy and outreach are yet to be explored and resolved before such research targeting cancer stem cells can enter clinical trials.

Further elucidation of the biological features of these putative tumor initiating cells with radical survival capacity and tumorigenic potential may impart new insights on factors that drive sudden acquired drug resistance. This survival tactic of cancer cells has been consistently creating numerous hurdles towards restorative therapy for a number of human malignancies. Better comprehension of the concept and reassessment of existing preclinical drug development paradigms that target the molecular pathways controlling self renewal, survival, and resistance in cancer stem cells can significantly improve existing therapeutic inconsistencies by eliminating both the root and stem of the disease.

## Figures and Tables

**Table 1:** An account of stem cell-like SP phenotype in malignancies.

Tumor type	(%) of sp cells	Cellular phenotype and features of sp cells	Ref.
Breast	0.18 ± 0.45%	CD44+/CD24−/vimentin higher and lower levels of cytokeratins compared to non-SP cells.	[53]
			[54]

Lung adenocarcinoma	24%	SP cells showed chemoresistance to platinum drugs and high expression of genes related to drug resistance (AKR1C1/C2 and NR0B1) or cancer metastasis (TM4SF1) & high mRNA expression of ABCG2, ABCC2.	[55]

Endometrial cancer	0.02%	Higher expression levels of vimentin, alpha-smooth muscle actin, and collagen III in SP induced tumors.	[56, 57]

Ewing's sarcoma	1.2%	High clonogenicity, invasiveness, and ABC transporter expression in SP cells than non-SP	[58]

Glioblastoma	0.4–1.5%	CD133+ SP cells coexpressed nestin and generated tumors in brains of NOD/SCID mice.	[59]

Prostate cancer	0.1–0.9%	CD133+, CD45−, CD81+, Sca-1+ SP clonal cells secreted (TGF-beta1)	[60]

Pancreatic cancer	2.1–8.7%	CD133+ SP cells showed significant levels of mRNA expression for CD133, ABCG2, and Notch1 than non-SP cells.	[61]

Leukemia	0.008–4.1%	CD34(+) CD38(−) CD123+	[62]

Hepatocellular carcinoma	0.1–28.7%	SP cells showed high chemoresistance, self-renewal, clonogenicity, and ABCG2 expression.	[63]

Medulloblastoma	12.4–39.1%	CD133+ SP cells with increased cell size, decreased S-phase, and proliferative capacity.	[64]

Renal epithelial malignancy	5.9 ± 0.9%	SP enriched for quiescent cells with high proliferative capacity and stem-like properties.	[65]

Head & neck squamous Carcinoma	0.69–0.9%	Activation of EGFR, a gene implicated in HNSCC tumorigenesis leads to increased SP.	[32]

Urological malignancy	0.1–0.6%	SP fraction has enhanced colony forming and proliferative capacity.	[66]

Ovarian cancer	0.9%	SP cells showed higher levels of Oct3/4 and colony formation efficiency than non-SP cells.	[67]

Nasopharyngeal carcinoma	2.6%	CK19 highly expressed SP cells were more chemo/radiation resistant related to the expression of ABCG2/smoothened protein.	[68]

Melanoma	0.96%	CD44+/CD133+/CD24+/ABCG2 high SP cells.	[69]

Laryngeal cancer	1.7–17%	SP cells had high self-renewal, proliferation, radiation resistance, and tumorigenicity.	[70]

Gastric cancer	0.001–12%	High expression levels of adhesion molecules *α*2, *α*5, *β*3 and *β*5 integrins, CD44, Oct3/4, and Sox2 in SP cell-injected tumors.	[71]

**Table 2:** Multidrug resistance mediated by SP cells.

SP expressing cancers	Resistance to drugs	Ref.
Gastric cancer cell line SGC-7901	5-Fluorouracil cisplatin, 5-fluorouracil, doxorubicin	[18, 72]
Melanoma	Paclitaxel	[73]
Malignant pleural mesothelioma	Mitoxantrone	[50]
Head and neck squamous cell carcinoma	5-Fluorouracil mitoxantrone	[74, 75]
CLL	Fludarabine, bendamustine, or rituximab	[76]
Pancreatic carcinoma	Gemcitabine	[77, 78]
Nasopharyngeal	Mitoxantrone, cisplatin, mitomycin-C	[68]
Endometrial	Paclitaxel	[56]
Glioma	5-Fluorouracil and carboplatin	[79]
Breast	Paclitaxel, epirubicin, mitoxantrone, carboplatin	[80]
		[54]
Lung	Mitoxantrone, doxorubicin	[55]
Esophageal	5-Fluorouracil	[81]
Bladder	Mitomycin-C, mitoxantrone	[82]
